# Association between vitamin D plasma concentrations and *VDR* gene variants and the risk of premature birth

**DOI:** 10.1186/s12884-019-2671-2

**Published:** 2019-12-31

**Authors:** Letícia Veríssimo Dutra, Fernando Alves Affonso-Kaufman, Fernanda Ramires Cafeo, Milene Saori Kassai, Caio Parente Barbosa, Francisco Winter Santos Figueiredo, Fabíola Isabel Suano-Souza, Bianca Bianco

**Affiliations:** 1 Postgraduation Program in Health Sciences, Faculdade de Medicina do ABC, Santo Andre, Brazil; 2 Medical Student, Faculdade de Medicina do ABC, Santo Andre, Brazil; 3 Department of Pediatrics, Discipline of Pediatrics, Faculdade de Medicina do ABC, Santo André, Brazil; 4 Department of Collective Health, Discipline of Sexual and Reproductive Health and Populational Genetics, Faculdade de Medicina do ABC, Santo Andre, Brazil

**Keywords:** Genetic variant; Vitamin D; Premature birth; gestation; newborns.

## Abstract

**Background:**

Premature birth is the main cause of mortality in children under 1 year, and vitamin D deficiency during gestation is associated with prematurity. The effects of vitamin D are mediated by its receptor, which is encoded by the *VDR* gene. *VDR* variants—such as single nucleotide variation (SNV)—are associated with increased risk of prematurity, but there are conflicting results. We evaluated serum vitamin D concentrations and the frequency of TaqI/A > G, BsmI/C > T, ApaI/C > A, and FokI/A > T *VDR* variants in mothers and preterm (PTN) and full-term (FTN) newborns.

**Methods:**

We conducted a case-control study comprising 40 pairs of mothers and their PTNs (gestational age < 32 weeks and/or weight < 1500 g), and 92 pairs of mothers and FTNs as controls. Genotyping was performed by real-time PCR, and plasma vitamin D concentrations were measured by electrochemiluminescence.

**Results:**

Vitamin D levels were significantly lower in PTN mothers. Genotypes TaqI/GG and BsmI/TT, and haplotypes AAG (TaqI/A-ApaI/A-FokI/G) and GCA (TaqI/G-ApaI/C-FokI/A) were significantly more frequent in PTN mothers, and genotypes TaqI/AG, ApaI/AA, and FokI/AG resulted in significantly lower vitamin D levels. Genotypes BsmI/TT and ApaI/AA were associated with vitamin D deficiency and 2.36 and 7.99 times greater likelihood of PTB, respectively. Vitamin D levels were also lower in PTNs, although it was not statistically significant. Genotypes BsmI/TT, ApaI/AA, and FokI/GG, and haplotype GAG (TaqI/G-ApaI/A-FokI/G) were significantly more frequent in PTNs. Those with FokI/GG genotypes had significantly lower vitamin D levels.

**Conclusions:**

*VDR* variants contribute to variations in vitamin D concentrations and the increased risk of prematurity.

## Introduction

A preterm birth is defined as a delivery that occurs before 37 weeks’ gestation, and can be classified as late preterm (36–34 weeks), very preterm (28–34 weeks), or extremely preterm (less than 28 weeks) [1, 2]. Prematurity is the main cause of death among children under 5 years of age [3], and preterm infants may present with early and late health complications involving the immune, respiratory, cardiovascular, gastrointestinal, and endocrine systems, as well as growth and developmental problems [3, 4]. The estimated overall global premature birth rate is 11.1% [5], and with an approximate rate of 11.8% [6], Brazil is in the top 10 countries with the highest rates [1, 2] .

Studies have associated prematurity with low serum levels of vitamin D during gestation, which is a period of risk for vitamin D deficiency and insufficiency. Vitamin D can be obtained in several ways: by endogenous synthesis in the skin driven by exposure to ultraviolet B rays in sunlight, during which 7-dehydrocholesterol is converted to cholecalciferol (vitamin D3) [7]; and from dietary sources such as fatty fish, which contain cholecalciferol [8], and plants and fungi containing ergocalciferol (vitamin D2) [9]. Both vitamin D2 and vitamin D3 undergo two hydroxylation reactions to become active: the first in the liver by 25-hydroxylase, which produces 25(OH) D (the circulating form); and the second in the kidneys by 1α-hydroxylase, which produces 1,25(OH)2D (the metabolically active form) [10].

An adequate level of vitamin D 25(OH) D is above 30 ng/mL (75 nmol/L); 21–29 ng/mL (51–74 nmol/L) is insufficient, and below 20 ng/mL (50 nmol/L) is low [11]. The global mean of vitamin D deficiency is 29.8% and the global mean of insufficiency during gestation is 87.0%. Vitamin D deficiency during pregnancy is associated with increased risk of gestational diabetes, preeclampsia, fetal growth restriction, bacterial vaginosis, caesarean section, and prematurity [12]. Vitamin D deficiency is also common among infants in several countries; prevalence ranges from 50 to 70% in the United States [13], and it is approximately 60.5% in Brazil [14].

Most of the biological activities attributable to vitamin D are mediated by its receptor (VDR), which is encoded by the *VDR* gene (*vitamin D receptor*; Gene ID 7421; MIM 601769) with a chromosomal locus of 12q13.11. It is a member of the steroid hormone receptor family that mediates the action of vitamin D by regulating the transcription of multiple genes [15]. Alterations in the *VDR* gene can lead to important defects in gene activation that can affect calcium metabolism [10], cell proliferation [8, 16], and immune function [8]. The most studied *VDR* gene single nucleotide variation (SNV) related to clinical outcomes are ApaI, BsmI, FokI, and TaqI [17–22].

Studies have produced conflicting results regarding the association between *VDR* gene variants and prematurity [17–22]. Different allelic frequencies among the populations arise from different genetic ancestries, so the racial origins of the studied population may be responsible for the divergent results. Hence, Brazilian population shows contributions from three main parental groups: Amerindian, European, and African [23, 24], this heterogeneity can produce different allele frequencies from those presented by non-mixed populations.

In the present study, we investigated the possible influence of vitamin D plasma concentrations and the frequency of *VDR* gene TaqI, BsmI, ApaI, and FokI variants in mothers and their preterm and full-term newborns.

## Methods

### Subjects

We carried out a case-control study involving 132 mothers in puerperia and their newborns at the Hospital Municipal Universitário de São Bernardo do Campo of the Faculdade de Medicina do ABC, Santo André, Brazil, who were recruited from September 2016 to December 2017.

The participants were divided into 2 groups: a case group comprising 40 pairs of mothers and their preterm newborns (PTNs); and a control group comprising 92 pairs of mothers and their full-term newborns (FTNs). Newborns that had gestated for less than 32 weeks and/or had birth weights of less than 1500 g were included in the case group, and the control group contained newborns that had gestated for 37 to 41 6/7 weeks. The classification of birth weight and gestational age was in accordance with the INTERGROWTH-21 consortium [25, 26]. The exclusion criteria were: newborns with major malformations, genetic syndromes, and severe neonatal anoxia, and cases where it was impossible to measure vitamin D levels.

The data and sample collection were only performed after all participants had signed written consent to participate, approved by the ethics committee of the Faculdade de Medicina do ABC (CAAE: 54166216.5.0000.0082).

### Sample collection

At the time of delivery, 10 mL of peripheral blood was collected from each mother by peripheral venipuncture in a tube containing a clot-separating gel and a tube containing ethylenediaminetetraacetic acid (EDTA). We collected 10 mL of umbilical cord blood from the PTNs at the time of delivery in a tube containing clot-separating gel and exfoliated cells from the oral mucosa after discharge. After collection, the tubes for the biochemical dosages were centrifuged (1500 rpm for 10 min), and the plasma was aliquoted into microtubes and frozen at − 20 °C. The tube for DNA extraction was stored in a refrigerator at 8 °C until required.

### Vitamin D

We determined the levels of 25(OH) D by electrochemiluminescence using an Elecsys® Vitamin D Total II kit (Roche, Basel, Switzerland). The requisite level of 25(OH) D is considered to be > 30 ng/mL [27].

### Genotyping

We extracted DNA from each mother’s peripheral blood lymphocytes using the salt-out method described by Lahiri and Nurnberger [28], and from the oral mucosal cells of newborns using an ORAcollect® DNA OCR-10 kit (Genotek, Ontario, Canada) according to the manufacturer’s instructions. The *VDR* genotypes of the TaqI (NC_000012.11:g.48238757A > G, rs731236), BsmI (NC_000012.11:g.48239835C > T, rs1544410), ApaI (NC_000012.11:g.48238837C > A, rs7975232), and FokI (NC_000012.11:g.48272895A > G, rs2228570) variants were identified by real-time PCR using TaqMan probes from Thermo Fisher Scientific® (Waltham, Massachusetts, USA) according to the manufacturer’s instructions, and a StepOne™ Real-Time PCR System (Applied Biosystems™, Thermo Fisher Scientific®, Massachusetts, USA).

Single Nucleotide Variants were described according to Human Genome Variation Society (HGVS) guidelines (http://www.hgvs.org/content/guidelines) [29].

### Statistical analyses

We performed the statistical analyses using Stata® software (SE 11.0) for Windows. The data were described by absolute and relative frequencies, and by assessing central tendency and dispersion. We used the Shapiro–Wilk test to determine the data’s normality. The qualitative variables were analyzed using the chi-square test, the Mann–Whitney test was used for the continuous variables without normal distributions, and Student’s *t* test was used for quantitative variables with normal distributions.

The chi-square test was also used to detect differences in allele and genotype frequencies of the *VDR* variants between groups, and to estimate the Hardy–Weinberg equilibrium (HWE). The associations between the combined alleles of *VDR* variants and the prematurity risk were evaluated using Haploview software version 4.1, available at http://www.hapmap.org.

Odds ratios (ORs) and confidence intervals (CIs) were used to measure the strength of the associations among the frequencies of *VDR* genotypes, vitamin D concentration, and prematurity. All *P*-values were two-tailed, and 95% CIs were calculated. The associations between genotypes of the *VDR* gene variants, isolated and adjusted according to vitamin D concentrations, with prematurity risk were calculated by logistic regression. *P*-values < 0.05 were considered statistically significant.

## Results

The data relating to the mothers and PTN and FTN newborns are shown in Table 1. The mean age of the mothers who experienced preterm births was 26.4 ± 7.1 years, compared to 25.8 ± 6.9 years for mothers who experienced full-term births. The mean pre-gestational body mass index for the mothers of PTNs was 27.2 ± 4.9 kg/m^2^, compared to 26.2 ± 5.7 kg/m^2^ for the mothers of FTNs. Two mothers (5.0%) in the case group and three (3.3%) mothers in the control group received 25(OH) D supplementation during gestation (*P* = 0.631). Considering pregnancy diseases, specific hypertension disease in pregnancy was found in 45.0% of mothers in the case group and 13.0% in the control group (*P* < 0.001); however, gestational diabetes mellitus (5.0% versus 2.2%) and urinary tract infection (22.5 and 33.7%) were not statistically different between groups. Only 10.0% of mothers in the case group reported regular solar exposure compared with 66.3% of mother of the control group (*P* < 0.001).

**Table 1:** Characteristics of mothers and their preterm and full-term newborns

Variables	Preterm	Full-term	*P-value*
Mothers			
Age (years) (*n* = 129)	26.4 ± 7.1	25.8 ± 6.9	0.615^1^
Pre-gestational body mass index (kg/m^2^) (*n* = 108)	27.2 ± 4.9	26.2 ± 5.7	0.458^1^
Education (*n* = 128)			
0–4 years	2 (5.3%)	2 (2.2%)	0.263^2^
4–8 years	30 (78.9%)	63 (70.0%)	
8–12 years	6 (15.8%)	25 (27.8%)	
Alcoholism (*n* = 132)	2 (5.0%)	5 (5.4%)	0.918^2^
Smoking (*n* = 132)	5 (12.5%)	10 (10.9%)	0.786^2^
Type of birth (*n* = 132)			
Vaginal	12 (30.0%)	40 (43.5%)	0.191^2^
cesarean section	28 (70.0%)	50 (54.3%)	
Fórceps	0 (0.0%)	2 (2.2%)	
Pregnancy diseases (*n* = 132)			
Specific hypertension disease in pregnancy	18 (45.0%)	12 (13.0%)	< 0.001^2^
Gestational diabetes mellitus	2 (5.0%)	2 (2.2%)	0.384^2^
Urinary tract infection	9 (22.5%)	31 (33.7%)	0.198^2^
Pregnancies’ number (*n* = 132)	1.95 ± 1.15	2.2 ± 1.3	0.243^1^
Vitamin D Supplementation (*n* = 132)	2 (5.0%)	3 (3.3%)	0.631^2^
Folic acid (yes) (*n* = 132)	32 (80.0%)	81 (88.0%)	0.226^2^
Iron (yes) (*n* = 132)	33 (82.5%)	78 (84.8%)	0.742^2^
Photo protection frequently (*n* = 132)	8 (20.0%)	14 (15.2%)	0.498^2^
Regular solar exposure (*n* = 132)	4 (10.0%)	61 (66.3%)	< 0.001^2^
Vitamin D (ng/ml) (*n* = 126)	20.8 ± 11.8	26.5 ± 9.7	0.004^1^
Sufficent levels (> 30 ng/mL)	7 (17.5%)	35 (40.7%)	0.036^1^
Insufficient levels (21–29 ng/mL)	14 (35.0%)	23 (26.7%)	
Deficient levels (≤ 20 ng/mL)	19 (47.5%)	28 (32.6%)	
Newborns			
Gestational age (weeks) (*n* = 131)	29.6 ± 2.5	39.1 ± 1.8	< 0.001^1^
Birth weight (g) (*n* = 131)	1239 ± 336	3285 ± 558	< 0.001^1^
Birth length (cm) (*n* = 94)	38.7 ± 2.8	48.3 ± 3.1	< 0.001^1^
Birth cephalic perimeter (cm) (*n* = 112)	26.9 ± 2.1	34.3 ± 1.7	< 0.001^1^
Gender (*n* = 130)			
Male	17 (43.6%)	51 (56.0%)	0.193^2^
Female	22 (56.4%)	40 (44.0%)	
Gestational Age classification^a^ (*n* = 130)			
Small for gestational age	7 (17.5%)	4 (4.4%)	0.037^1^
Adequate for gestational age	29 (72.5%)	71 (78.9%)	
Large for gestational age	4 (10.0%)	15 (16.7%)	
Vitamin D (ng/ml) (*n* = 130)	27.2 ± 13.4	31.7 ± 11.7	0.056^1^
Sufficent levels (> 30 ng/mL)	16 (42.1%)	47 (56.0%)	0.064
Insufficient levels (21–29 ng/mL)	9 (23.7%)	24 (28.6%)	
Deficient levels (≤ 20 ng/mL)	13 (34.2%)	13 (15.4%)	

^1^T test; ^2^ Chi-square test

### Vitamin D

The plasma concentrations of 25(OH) D in the mothers and newborns are shown in Table 1. Samples were collected in the spring and summer seasons for 88.6% of the cases, including 100.0% of the full-term group (25(OH) D mean 31.7 ± 11.7 ng/mL) and 62.5% of the premature group (25(OH) D mean 24.5 ± 12.9 ng/mL), whereas 11.4% were collected in the autumn and winter seasons, comprising 37.5% (25(OH) D mean 31.2 ± 13.6 ng/mL) of the preterm group.

With regard to the plasma concentrations of 25(OH) D in the mothers, 7 (17.5%) of the PTN mothers and 35 (40.7%) of the FTN mothers presented with sufficient vitamin levels (≥ 30 ng/mL); 14 (35.0%) of the PTN mothers and 23 (26.7%) of the FTN mothers presented with insufficient levels (21–29 ng/mL); and 19 (47.5%) of the PTN mothers and 28 (32.6%) of the FTN mothers had deficient plasma concentrations (≤ 20 ng/mL) (*P* = 0.036).

With regard to the plasma concentrations of 25(OH) D in the newborns, 16 (42.1%) of the PTNs and 47 (56.0%) of the FTNs had sufficient 25(OH) D levels; 9 (23.7%) of the PTNs and 24 (28.6%) of the FTNs had insufficient levels; and 13 (34.2%) of the PTNs and 13 (15.4%) of the FTNs were deficient (*P* = 0.064).

### *VDR* gene variants

With regard to the *VDR* variants, the genotype and allele frequencies of the TaqI, BsmI, ApaI, and FokI variants in the PTN and FTN mothers and newborns are shown in Table 2. The frequencies of the studied variant genotypes were in HWE, except for the BsmI SNV in the mothers and their PTNs.

**Table 2:** The genotype and allele frequencies of the TaqI, BsmI, ApaI, and FokI variants of the *VDR* gene in preterm and full-term mothers and newborns

*VDR* SNVs	Population	N	Genotypes					HWE	Alleles		
			n (%)	n (%)	p	n (%)	p		n (%)	n (%)	p
Mothers											
			**AA**	**AG**		**GG**			**A**	**G**	
TaqI/A > G	Preterm	40	19 (47.5)	18 (45.0)	0.105	3 (7.5)	0.009	0.903	56 (70.0)	24 (30.0)	0.007
	Full-term	92	25 (27.2)	46 (50.0)		21 (22.8)		1.0	96 (52.2)	88 (47.8)	
			**CC**	**CT**		**TT**			**C**	**T**	
BsmI/C > T	Preterm	40	15 (37.5)	0	0.001	25 (62.5)	0.031	< 0.001	30 (37.5)	50 (62.5)	0.005
	Full-term	92	34 (37.0)	35 (38.0)		23 (25.0)		0.260	103 (56.0)	81 (44.0)	
			**CC**	**CA**		**AA**			**C**	**A**	
ApaI/C > A	Preterm	40	5 (12.5)	21 (52.5)	0.067	14 (35.0)	< 0.001	0.799	31 (38.8)	49 (61.2)	0.001
	Full-term	92	31 (33.7)	49 (53.3)		12 (13.0)		0.558	111 (60.3)	73 (39.7)	
			**AA**	**AG**		**GG**			**A**	**G**	
FokI/A > G	Preterm	40	4 (10.0)	18 (45.0)	0.284	18 (45.0)	0.323	0.987	26 (32.5)	54 (67.5)	0.436
	Full-term	92	16 (17.4)	37 (40.2)		39 (42.4)		0.397	69 (37.5)	115 (62.5)	
Newborns											
			**AA**	**AG**		**GG**			**A**	**G**	
TaqI/A > G	Preterm	40	17 (42.5)	18 (45.0)	0.788	5 (12.5)	0.447	1.0	52 (65.0)	28 (35.0)	0.705
	Full-term	92	39 (42.4)	46 (50.0)		7 (7.6)		0.419	124 (67.5)	60 (32.5)	
			**CC**	**CT**		**TT**			**C**	**T**	
BsmI/C > T	Preterm	40	14 (35.0)	1 (2.5)	0.002	25 (62.5)	0.001	< 0.001	29 (36.3)	51 (63.7)	< 0.001
	Full-term	92	38 (41.3)	37 (40.2)		17 (18.5)		0.348	113 (61.4)	71 (38.6)	
			**CC**	**CA**		**AA**			**C**	**A**	
ApaI/C > A	Preterm	40	6 (15.0)	16 (40.0)	0.839	18 (45.0)	0.004	0.900	28 (35.0)	52 (65.0)	0.002
	Full-term	92	23 (25.0)	55 (59.8)		14 (15.2)		0.139	101 (54.9)	83 (45.1)	
			**AA**	**AG**		**GG**			**A**	**G**	
FokI/A > G	Preterm	40	1 (2.5)	19 (47.5)	0.109	20 (50.0)	0.037	0.358	21 (26.3)	59 (73.7)	0.053
	Full-term	92	12 (13.0)	47 (51.1)		33 (35.9)		0.757	71 (38.6)	113 (61.4)	

*SNV* Single Nucleotide Variant described according to HGVS nomenclature, *OR* Odds Ratio;

*CI* Confidence Interval, *HWE* Hardy-Weinberg Equilibrium

*Chi-square test. Wild genotype as reference. *P* < 0.05

With regard to the mothers, the frequencies of the GG variant genotype of TaqI SNV (22.8 and 7.5% for the full-term and preterm groups, respectively) and the CT heterozygous genotype of BsmI SNV (38.0 and 0%) were significantly higher in the full-term group than in the preterm group, whereas the TT variant homozygous genotype of BsmI SNV was significantly more frequent in the PTN mothers (62.5 and 25.0%). We observed similar genotype frequencies with regard to the BsmI SNV in the newborns. The frequency of the CT heterozygous genotype was significantly higher in the FTNs (40.2 and 2.5%), whereas the TT variant homozygous genotype was more frequent in the PTNs (62.5 and 18.5%). The AA variant homozygous genotypes of ApaI SNV was also significantly more frequent among the PTNs mother (35 and 13%) and preterm newborns (45.0 and 15.2%). The GG genotype of FokI SNV (50.0 and 35.9%) were also significantly more frequent among the PTNs.

The associations of the *VDR* gene TaqI, ApaI, and FokI combined allele in the mothers and their PTNs and FTNs are shown in Table 3. Because the BsmI variant was not in HWE in the mothers and PTNs, we did not consider this SNV in the haplotype analysis. The AAG (TaqI/A-ApaI/A-FokI/G) (33.5 and 20.4% in the PTN and FTN groups, respectively) and GCA (TaqI/G-ApaI/G-FokI/A) (2.7 and 1.1%) haplotypes were more frequent among the PTN mothers, whereas the GCG (TaqI/G-ApaI/C-FokI/G) haplotype was more frequent in the FTN mothers (22.5 and 5.8%). Among the newborns, the GAG (TaqI/G-ApaI/A-FokI/G) (18.5 and 5.6%) haplotypes was more frequent in the PTNs, whereas the GCA (13.5 and 4.0%) haplotype was more frequent in the FTNs.

**Table 3:** Haplotype analysis of TaqI, ApaI, and FokI variants of the *VDR* gene in mothers and their preterm and full-term newborns

Population	Haplotypes			Preterm	Full-term	P*
	TaqI (A > G)	ApaI (C > A)	FokI (A > G)	Frequency (%)	Frequency (%)	
Mothers						
	A	A	G	33.5	20.4	0.022
	G	C	G	5.8	22.5	0.001
	A	C	A	14.5	14.8	0.947
	A	C	G	15.8	12.0	0.395
	G	A	G	12.5	7.7	0.213
	G	C	A	2.7	1.1	0.025
	G	A	A	9.1	6.6	0.477
	A	A	A	6.2	5.0	0.687
Newborns						
	A	A	G	34.6	26.7	0.188
	A	C	G	17.3	20.1	0.595
	A	C	A	10.4	12.2	0.670
	G	C	A	4.0	13.5	0.021
	G	A	G	18.5	5.6	0.001
	G	C	G	3.3	9.1	0.099
	A	A	A	2.7	8.4	0.085
	G	A	A	9.2	4.4	0.131

*Software Haploview version 4.1. *P* < 0.05

### Genotypes of *VDR* variants and 25(OH) D concentration

The concentrations of 25(OH) D in relation to the genotypes of *VDR* gene variants in the mothers and newborns are presented in Table 4. The PTN mothers with the AG genotype of the TaqI SNV, the AA genotype of the ApaI SNV, and the AG genotype of the FokI SNV had significantly lower 25(OH) D levels than the FTN mothers. With regard to PTNs, the carriers of the GG genotype of the FokI SNV had significantly lower 25(OH) D levels than the FTN group.

**Table 4:** The relationships between the concentrations of 25(OH) D and the genotypes of the *VDR* gene variants in mothers and their preterm and full-term newborns

*VDR* SNVs	Genotypes	N	Preterm		N	Full-term		*P* **
			%	Vitamin D* (ng/mL)		%	Vitamin D* (ng/mL)	
Mothers (*n* = 132)								
TaqI/A > G	AA	19	47.5	20.90 (16.18; 25.62)	25	27.2	24.74 (20.025 28.09)	0.291
	AG	18	45.0	20.83 (13.79; 27.87)	46	50.0	28.05 (25.010; 30.96)	0.023
	GG	3	7.5	19.90 (−10.42; 50.22)	21	22.8	25.24 (21.30; 29.17)	0.348
BsmI/C > T	CC	15	37.5	21.54 (16.17; 26.91)	34	37.0	26.48 (22.77; 30.19)	0.131
	CT	0	0.0	–	35	38.0	28.24(24.75; 31.73)	***
	TT	25	62.5	20.35 (14.95; 25.75)	23	25.0	23.93 (21.00; 26.87)	0.245
ApaI/C > A	CC	5	12.5	20.28 (14.26; 26.30)	31	33.7	24.07 (20.71; 27.43)	0.375
	CA	21	52.5	23.71 (17.47; 29.96)	49	53.3	28.04 (25.27 30. 80)	0.136
	AA	14	35.0	16.60 (11.17; 22.04)	12	13.0	26.60 (19.80; 33. 40)	0.018
FokI/A > G	AA	4	10.0	20.71 (−9.45; 50.88)	16	17.4	26.61 (22.08; 31.13)	0.348
	AG	18	45.0	20.26 (14.97; 25.55)	37	40.2	26.96 (23.77; 30.17)	0.022
	GG	18	45.0	21.34 (15.41; 27.28)	39	42.4	26.04 (22,68; 29.40)	0.136
Newborns (*n* = 38)								
TaqI/A > G	AA	18	47.4	27.95 (21.13 34.78)	39	42.4	30.436 (27.510; 33.76	0.451
	AG	16	42.1	27.64 (20.14; 35.15)	46	50.0	32.76 (28.93; 36.59)	0.186
	GG	4	10.5	21.58 (5.42; 37.74)	7	7.6	31.60 (20.60; 42.59)	0.192
BsmI/C > T	CC	14	36.8	28.68 (20.09; 37.27)	38	41.3	31.78 (27.85;35.72)	0.441
	CT	1	2.6	39.62	37	40.2	33.37 (42)	***
	TT	23	60.6	25.68 (20.21; 31.15)	17	18.5	27.80 (22.87; 32.73)	0.566
ApaI/C > A	CC	6	15.7	26.78 (15.15; 38.40)	23	25.0	33.31 (26.88; 39.74)	0.326
	CA	15	39.5	32.04 (23.34; 40.75)	55	59.8	31.74 (28.70; 34.79)	0.934
	AA	17	44.8	22.97 (17.37; 28.57)	14	15.2	28.79 (24.99; 32.60)	0.090
FokI/A > G	AA	1	2.6	37.66	12	13.0	34.17 (25.64; 42.70)	***
	AG	19	50.0	30.00 (23.72; 36.27)	47	51.1	30.76 (27.75; 33.77)	0.801
	GG	18	47.4	23.57 (18.87; 30.26)	33	35.9	32.10 (27.44; 36.76)	0.032

N: Sample number. * Mean concentrations of 25(OH) D (confidence interval). **T-test. *** Not possible calculated due sample size. *P* < 0.05

### Prematurity risk

The risks of prematurity in relation to the genotypes of *VDR* variants associated with 25(OH) D deficiency in the mothers are shown in Table 5. Regardless of their association with 25(OH) D deficiency, the variant genotypes of the BsmI/TT and ApaI/AA SNVs were associated with the increased risk of prematurity. Mothers with the TT variant genotype of the BsmI SNV, and with 25(OH) D deficiency, had a 2.36-fold increased chance of preterm birth, whereas carriers of the variant genotype AA of the ApaI SNV had an increased risk of prematurity, i.e., 7.99-fold higher. The carriers of the GG genotype of the TaqI SNV had a lower risk of preterm birth, even when associated with 25(OH) D deficiency.

**Table 5:** The risks of prematurity in relation to the genotypes of the *VDR* variants, and their association with 25(OH) D deficiency in the mothers

*VDR* SNVs	Genotypes	Odds	Prematurity Risk			
			All mothers (*n* = 132)		Mothers with 25(OH) D deficiency (*n* = 47)	
			OR (CI 95%)	p*	OR (IC 95%)	p**
TaqI/A > G	AA	0.76	Ref.		Ref.	
	AG	0.39	0.51 (0.23; 1.16)	0.107	0.56 (0.25; 1.27)	0.165
	GG	0.14	0.19 (0.05; 0.72)	0.015	0.19 (0.05; 0.74)	0.016
BsmI/C > T	CC	0.44	Ref.		Ref.	
	CT	0.00	***	***	***	***
	TT	1.08	2.46 (1.07; 5.65)	0.033	2.36 (1.02 5.47)	0.044
ApaI/C > A	CC	0.16	Ref.		Ref.	
	CA	0.43	2.66 (0.91; 7.78)	0.075	3.00 (1.003; 8.99)	0.050
	AA	1.17	7.23 (2.14; 24.48)	0.001	7.99 (2.29; 27.84)	0.001
FokI/A > G	AA	0.25	Ref.		Ref.	
	AG	0.49	1.95 (0.57; 6.67)	0.290	2.01 (0.58; 7.02)	0.271
	GG	0.30	1.85 (0.54; 6.31)	0.329	1.82 (0.53; 6.33)	0.344

*SNV* Single Nucleotide Variant, *OR* Odds Ratio, *CI* Confidence interval. Wild genotype was used as a reference for *Logistic regression

** Logistic regression adjusted for maternal vitamin D deficiency (≤30 ng/ml). *** Not possible to calculate due to sample size. *P* < 0.05

## Discussion

During gestation, the mother is the fetus’ only source of vitamin D, which is transmitted across the placenta [30]. The levels of vitamin D in the mother are directly related to those in the fetus, and vitamin D deficiency has been associated with an increased risk of certain childhood diseases, such as rickets, infectious diseases, noncommunicable chronic diseases, obesity, and asthma [13]. The reduction in the level of albumin and the increase in the hepatic production of vitamin D-binding protein (DBP) during pregnancy alter maternal vitamin D metabolism. This reduces the availability of free vitamin D and increases the conversion of 25(OH) D to 1,25(OH)2D3 through the placenta, which is the main extra-renal site of this conversion owing to the increased activity of 1α-hydroxylase [30]. Moreover, the late transfer of vitamin D to the fetus may be impaired in preterm birth, which has prompted various investigations of serum vitamin D levels in pregnant women [31, 32].

A meta-analysis including 10,098 patients from 10 studies found an increased risk of preterm birth for pregnant women with vitamin D deficiency (< 20 ng/mL; OR = 1.29; 95% IC = 1.16–1.45) [31]. A meta-analysis performed by Zhou et al. (2017) [33] including 6 randomized controlled trials and 18 observational studies, the increased risk of prematurity was associated to deficiency (OR = 1.25; 95% IC = 1.13–1.38) more than maternal vitamin D insufficiency (OR = 1.09; 95% IC = 0.89–1.35). Recently, the association between maternal vitamin D deficiency and preterm birth was evaluated in a study by Woo et al. [34] comprising studies from 2012 to 2018, being non conclusive despite analysis of research indicates that vitamin D deficiency is related to an increased risk for preterm birth [34].

The evidence suggests that preterm labor is a heterogeneous condition with many triggering and precipitating factors, especially infections and inflammatory causes [35]. 1,25(OH)2D promotes cytokine inhibition and the expression of potent antimicrobial peptides in various immune cells, such as macrophages and dendritic cells, and acts on placental tissue by modulating anti-inflammatory effects [31].

Preeclampsia is a risk factor for premature birth whose only treatment is birth [36, 37]. A large body of evidence indicates that increased inflammatory response is a feature of systemic vascular dysfunction in this disorder, although its cause is not completely known [38, 39]. Due to vitamin D anti-inflammatory effects, it may play a role in preeclampsia prevention [34, 40–42]. A recent study [43] pointed that down-regulation of VDR expression and vitamin D deficiency may contribute to phenotypic changes of inflammatory patterns in maternal vasculature in preeclampsia. Otherwise, the *VDR* FokI variant was associated with decreased risk of preeclampsia in the dominant model in Iranian population [44].

Despite the evidence of an association between vitamin D deficiency and preterm birth and preeclampsia, data on the effect of vitamin D supplementation on preterm birth have not been consistent, probably due to the heterogeneity of studies regarding the differences in assay methodologies, definitions of vitamin D deficiency, timing of supplementation, and dose of supplementation [34]. A recent Cochrane review [39] showed evidence from 3725 pregnant women enrolled in 22 studies, suggesting that vitamin D supplementation alone during pregnancy potentially attenuates the risk of preeclampsia in comparison to placebo or no intervention. Also, it may have small or even no difference for the risk of premature birth. Other review raised evidence from nine studies involving 1916 pregnant women and suggested that the risk of preeclampsia is reduced when supplementation with vitamin D and calcium is performed, although may increases risk of preterm birth. Other than that, the benefits or harms of vitamin D supplementation alone or combined with calcium and other vitamins and minerals during pregnancy for mother and children remains unclear [39].

Currently, some countries and international scientific organizations recommend screening in pregnant women who are at risk for vitamin D deficiency and supplementation with doses ranging from 600 to 1000 IU/day [45–47]. In Brazil there are no recommendation for vitamin D screening and supplementation during pregnancy. WHO [48] reported a need for more evidence for recommending vitamin D supplementation greater than 200 units per day.

It is important to note that there are several other pregnancy and neonatal complications associated with maternal vitamin D deficiency such as increased incidence of gestational diabetes mellitus, low birth weight, and possible epigenetic effects on offspring [34].

In the present study, specific hypertension disease in pregnancy (45.0% versus 13.0%, respectively) was significantly higher in PTN mothers in relation to the FTN group; whereas gestational diabetes mellitus (5.0% versus 2.2%, respectively) and urinary tract infection (22.5% versus 33.7%, respectively) were not significantly different between PTN and FTN mothers. In addition, no difference was found considering the supplementation of vitamin D, folic acid, and iron. Only 10% of PTN mothers regularly sunbathed in relation to 66.3% in FTN mothers; however the frequency of photo protection was not different between groups (20.0% in PTN mothers and in 15.2% of FTN mothers). Regarding the 25(OH) D levels, the present study revealed that the 25(OH) D levels in preterm mothers was significantly lower in relation to the full-term group, and there was vitamin deficiency in 47.5% of the PTN mothers. The 25(OH) D levels in preterm newborns was lower in relation to the full-term newborns, although this difference was not statistically significant. There was vitamin deficiency in 34.2% of the PTNs.

Considering the *VDR* variants, the findings of previous studies include strong indications of no association between *VDR* variants and the risk of prematurity (Table 6). Only six studies have associated *VDR* gene variants with the risk of preterm birth, and no study has associated these SNVs with serum vitamin D levels.

**Table 6:** Case-control studies associated with *VDR* gene variantss and the risk of prematurity

Study	Population Origin	Groups	*VDR* SNVs*	Conclusions
Manzon et al. (2013) [17]	Israel (Jewish)	33 caucasian mothers and their PTN (24–35 weeks gestation) 98 mothers and their FTN	TaqI BsmI ApaI FokI	The frequency of the FokI/C allele was significantly higher in mothers who had preterm births.
Cai et al. (2016) [18]	China	57 mothers who had PTN 84 mothers who had FTN	FokI	The FokI/FF genotype was associated as a risk factor for preterm birth.
Baczyńska-Strzecha et al. (2016) [19]	Poland	100 caucasian mothers who had PTN (22–36.6 weeks gestation) 99 mothers who had FTN	TaqI BsmI ApaI	There was no difference in the frequency of the genotypes individually, but the combination of the genotypes BsmI/bb-ApaI/AA-TaqI/TT and BsmI/BB-ApaI/aa-TaqI/tt were more frequent in mothers who had preterm birth.
Rosenfeld et al. (2017) [20]	Israel (Jewish)	146 caucasian mothers and their PTN (24–36 weeks gestation) 229 mothers and their FTN	TaqI BsmI ApaI FokI	The ApaI/AA genotype was associated with an increased risk of preterm birth.
Javorski et al. (2018) [21]	Brazil (Northeast)	104 mothers who had PTN 85 mothers who had FTN	FokI	The FokI/T allele was associated with a higher risk of preterm birth.
Barchitta et al. (2018) [22]	Italy	17 mothers and their PTN (< 37 weeks gestation) 187 mothers and their FTN	FokI	The FokI polymorphic genotype in mothers was associated with an increased risk for preterm birth.
This study	Brazil (Southeast)	40 mothers and their PTN (23–32 weeks gestation) 92 mothers and their FTN	TaqI BsmI ApaI FokI	The BsmI/TT and ApaI/AA genotype increased prematurity risk, regardless of vitamin D deficiency. Preterm newborns with FokI/GG genotypes had lower serum vitamin D concentrations.

*SNV* Single Nucleotide Variant, *PTN* preterm newborn, *FTN* full-term newborn, *PTB* preterm birth. Alleles according to HGVS nomenclature: TaqI/A > G; BsmI/ C > A or C > G or C > T; ApaI/C > A; and FokI/A > C or A > G or A > T

Manzon et al. (2014) [17] investigated 33 Caucasian Jewish Israeli mothers and their preterm newborns (24–35 weeks’ gestation), and 98 other mothers and their FTNs. They reported that the C allele of the Fokl SNV was more frequent, and the T allele of the TaqI SNV was less frequent, in preterm mothers. There was no association between the genotypes of the studied variants and preterm and full-term birth. Furthermore, Cai et al. (2016) [18] investigated 57 preterm and 84 full-term Chinese mothers, and reported that the FF genotype of the Fokl SNV was associated with an increased risk of preterm birth.

Baczyńska-Strzecha et al. (2016) [19] investigated 100 Polish preterm and 99 full-term mothers. They reported that the frequencies of the individual genotypes did not differ. However, the BsmI/bb-ApaI/AA-TaqI/TT and BsmI/BB-ApaI/aa-TaqI/tt genotype combinations were significantly more frequent in the preterm group, whereas the BsmI/Bb-ApaI/AA-TaqI/Tt and BsmI/BB-ApaI/Aa-TaqI/tt combinations reduced the risk of preterm birth. In contrast, in a study by Rosenfeld et al. (2017) [20] - which included 146 Israeli Jewish women and their preterm newborns, and 229 other women and their full-term newborns - the CC homozygous genotype of the ApaI SNV was associated with premature birth.

A Brazilian study by Javorski et al. (2018) [21] - which included 104 preterm and 85 full-term mothers—revealed that the T allele and TT wild genotype of the FokI SNV were more frequent in women who had preterm births. However, an Italian study by Barchitta et al. (2018) [22] - which comprised 17 pairs of mothers and their PTNs and 187 pairs of mothers and their FTNs - revealed that the Fokl variant genotype was associated with an increased risk for preterm birth.

In the present study, the TT genotype of the BsmI and AA genotype of the ApaI SNVs, and the AAG (TaqI/A-ApaI/A-FokI/G) and GCA (TaqI/G-ApaI/C-FokI/A) haplotypes were significantly more frequent in the PTN mothers, whereas the GG genotype of TaqI and the CT genotype of the BsmI SNVs, and the GCG (TaqI/G-ApaI/C-FokI/G) haplotype were more frequent in the FTN mothers. With regard to the relationship between 25(OH) D levels and the genotypes of the gene *VDR* variants, the PTN mothers with the AG genotype of the TaqI, the AA genotype of the ApaI, and the AG genotype of the FokI SNVs had significantly lower 25(OH) D levels. Mothers with the TT variant genotype of the BsmI and the AA genotype of the ApaI SNVs with 25(OH) D deficiency had an increased risk of preterm birth, whereas the carriers of the GG genotype of the TaqI SNV had a lower risk.

In the newborns, the TT genotype of the BsmI, the AA genotype of the ApaI, the GG genotype of the Fokl SNVs, and the GAG (TaqI/G-ApaI/A-FokI/G) haplotype was significantly more frequent in the PTN group, whereas the frequencies of the CT genotype of the BsmI SNV and the GCA (TaqI/G-ApaI/C-FokI/A) haplotype were significantly higher in the FTN group. The PTN carriers of the GG genotype of the FokI SNV had significantly lower 25(OH) D levels.

The genotypes of the BsmI variant deviated from HWE in the PTNs mothers and their newborns. Hardy–Weinberg disequilibrium may indicate genotyping errors, population stratification, or selection bias [49]. However, the studied variants were identified by qPCR using TaqMan probes, which is a validated and robust methodology. This finding may indicate a possible association between the marker locus and the risk of prematurity.

Conflicting results can be attributed to important differences between studies. Ethnicity is an important factor in SNV prevalence among different geographic populations, and is especially important in admixed populations such as the Brazilian population. Brazil is a country of continental dimensions; it covers 8,516,000 km^2^, and is inhabited by more than 200 million people. Brazilian population origins traces back to the former Amerindians and the main sources of immigration, specially western-european and African [50]. With regard to the five geographical regions of Brazil (North, Northeast, Central-West, Southeast, and South), ancestry from North Brazil is mostly amerindian, Northeast and Central-West mainly African, and Southern and Southeastern predominantly European. For decades, new immigrants and migrants from other parts of Brazil have flocked to Southeast Brazil, where intermarriage between individuals of different ancestries is very common [50]. This could explain the differences between the findings presented here - which are based on a population from the Southeast region of Brazil - and those of Javorski et al. (2018) [21] - which were also based on a Brazilian population, but from the Northeast region, although none of these studies evaluated the ancestral origin of the studied population.

Moreover, the variants studied in the different publications were not the same, and the genetic nomenclature used in some studies was diverse from described in the *Human Genome Variation Society* (HGVS) [29] - which hindered the interpretation of the results. Thus, the studied groups are heterogeneous, which may make their interpretation and comparison difficult.

Considering the potential limitations of the present study, the main concern could be considered the sample size. Nevertheless, this is the first study to associate serum vitamin D concentrations and *VDR* gene variants with the risk of prematurity (less than 32 weeks’ gestation). In addition, we observed that a maternal variable, specific hypertension disease in pregnancy was significantly more frequent in PTN mothers that can be a possible confounding factors and may influence the levels of 25(OH) D and risk of prematurity. Despite that, we observed that mothers carriers of the BsmI/TT and ApaI/AA SNVs, individually or associated with vitamin D deficiency, had an increased chance of preterm birth.

## Conclusions

*VDR* gene variants help explain the variations in the levels of 25(OH) D in relation to the risk of prematurity in the studied population. The variant genotypes of the BsmI/TT and ApaI/AA SNVs, individually or associated with vitamin D deficiency, were associated with the increased risk of prematurity. Preterm newborns with the GG genotype of the FokI SNV had lower serum 25(OH) D concentrations.

## Data Availability

The datasets generated and/or analysed during the current study are available in the Mendeley repository at https://data.mendeley.com/datasets/b69mpd2yhb/1. DOI: 10.17632/b69mpd2yhb.1.

## Abbreviations

BMI: Body mass index (BMI)
CI: Confidence interval
EDTA: Ethylenediaminetetraacetic acid
FTN: Full-term newborn
HWE: Hardy–Weinberg equilibrium
OR: Odds ratio
PTB: Preterm birth
PTN: Preterm newborn
SNV: Single nucleotide variation
VDR: Vitamin D receptor
Vitamin D: 25(OH)D
